# The role of knowledge, primary care and community engagement to improve breast-screening access for Pakistani women in the United Kingdom: A secondary analysis of a qualitative study

**DOI:** 10.1177/13558196231155824

**Published:** 2023-04-11

**Authors:** Hooran M Khattak, Victoria G Woof, David P French, Louise S Donnelly, Helen Ruane, Fiona Ulph, Nadeem Qureshi, Nasaim Khan, D Gareth Evans, Kathryn A Robb

**Affiliations:** 1 School of Social and Political Sciences, 3526 University of Glasgow, UK; 2 Division of Psychology & Mental Health, School of Health Sciences, 5292 University of Manchester, UK; 3 Nightingale & Prevent Breast Cancer Research Unit, 5293 Manchester University NHS Foundation Trust, UK; 4 Division of Psychology & Mental Health, School of Health Sciences, 5292 University of Manchester, UK; 5 NIHR School of Primary Care, School of Medicine, 12207 University Park, UK; 6 Department of Genomic Medicine, MAHSC, 5293 Manchester University NHS Foundation Trust, UK; 7 Nightingale and Prevent Breast Cancer Research Unit, 5293 Manchester University NHS Foundation Trust, UK and Medical Genetics and Cancer Epidemiology, Department of Genomic Medicine, MAHSC, University of Manchester, Manchester University NHS Foundation Trust, UK; 8 School of Health and Wellbeing, 3526 University of Glasgow, UK

**Keywords:** Breast screening, screening access, ethnic minorities

## Abstract

**Objective:**

Breast cancer incidence is rising among Pakistani women in the United Kingdom. However, uptake of breast screening remains low. This study aimed to improve access to breast screening for British-Pakistani women by exploring their knowledge of breast cancer and the role of primary care and community networks to support screening access amongst British-Pakistani women.

**Methods:**

We undertook a secondary qualitative analysis of 18 semi-structured interviews with British-Pakistani women from East Lancashire in the United Kingdom. Anonymized transcripts of the interviews were used for a thematic analysis.

**Results:**

Three themes were identified in the interviewees’ responses: (i) ‘Women’s knowledge of breasts and breast cancer’, which described how a cultural taboo exists around Pakistani women’s bodies and around breast cancer; (ii) ‘Role of primary care’, which detailed how General Practitioners can support informed decisions and offer a trusted and valued information source; (iii) ‘Community engagement’, which described the potential to disseminate breast-screening information through the whole community, including primary care providers, all family members and mosques.

**Conclusions:**

Our analysis suggested three main targets for future interventions to improve access to breast screening for British-Pakistani women: (i) co-produced strategies to increase knowledge of breasts and breast screening; (ii) greater collaboration with local General Practitioners to support women to make informed choices about screening; and (iii) community engagement involving General Practitioners and community leaders, to inform everyone – not just screening-age women – about breast cancer and screening.

## Introduction

Breast screening aims to detect cancer at an earlier and more treatable stage. It involves the use of an X-ray test, also known as a mammogram, to check each breast for a growth or lump too small to see or feel. In the United Kingdom (UK), the National Health Service Breast Screening Programme (NHSBSP) was established in 1988. The NHSBSP invites women aged 50 to 70, once every three years, for a free breast screening. The programme saves approximately 1300 lives per year among those invited.^1^

A major driver of success of the NHSBSP is uptake, with approximately 70% of eligible women attending in England.^2^ However, uptake of the NHSBSP is consistently low in Pakistani-dense areas in the UK.^3,4^ There are approximately 1.1 million Pakistanis in the population of England and Wales, and 49% of this population identifies as female. Approximately 9.9% of the total Pakistani population is between the ages of 50–70, making low uptake of screening a significant public health challenge.^5^

There is a perception amongst some women of South Asian ethnicity (including Pakistani, Indian, Bangladeshi, Afghani, Nepalese, Bhutanese, Maldivian and Sri Lankan) that they are at lower risk of developing breast cancer compared to White women, but this view is not in accordance with evidence.^6,7^ A study in the Greater Manchester area found no significant difference in incidence of breast cancer based on ethnicity.^7^ Ethnic differences in breast cancer risk between South Asian and Black women in comparison to White women were mostly accounted for by known risk factors such as height, age at menarche, childbearing and breastfeeding history, consumption of alcohol, and use of hormone replacement therapy. Once these risk factors were adjusted for, there were no differences in incidence by ethnic group.^6^ Asian women tend to have lower risks of breast cancer in their native countries; however, the move to a country with higher incidence results in gradually increasing levels of risk.^4^ This higher incidence is attributed to adopting new westernized lifestyle factors, such as reduced levels of physical activity, increased alcohol consumption and having fewer children, later in life.^6,7^

Given this increasing breast cancer incidence but low uptake of screening, British-Pakistani women’s access to the NHSBSP needs to be understood and addressed. Szczepura suggested that to reduce inequalities in access to a healthcare service for ethnic minority populations, intrinsic barriers to access need to be addressed.^8^ These include, but are not limited to, cultural differences, beliefs, suitable methods of dissemination of knowledge, language and tackling unfamiliarity due to the ‘newness’ of a concept.^8^

The cultural taboo surrounding breast cancer complicates engagement with awareness programmes and informational materials – privacy and modesty are valued strongly by Pakistani women, making the breasts and the female body, overall, a taboo to expose or even talk about.^8,9^ Saeed et al. identified individual, sociocultural and structural barriers in accessing information and screening, all of which prevent Pakistani women to seek timely diagnosis.^10^ Culturally, there is fear of social isolation as there is a belief amongst some Pakistani communities that those diagnosed with breast cancer are ‘unclean’ and should be avoided.^10^

Karbani et al. conducted a qualitative study involving interviewing 24 South Asian breast cancer patients, including 12 Pakistani women, in West Yorkshire to better understand cultural practices regarding breast cancer. Seven of the interviews had to be conducted outside the women’s homes because the women found it ‘restricting to talk about their illness’ inside their own houses.^11^

Lack of engagement with breast screening and delay in help-seeking are also suggested to be interlinked to belief systems. Some Pakistani Muslim women with low awareness reportedly deem a cancer diagnosis as ‘God’s will’ and look to spiritual healers for their treatment.^10,11^

British-Pakistanis and other ethnic minority populations lack awareness of cancer-screening programmes in the UK.^12^ Evidence from diabetes management, and in help-seeking for memory problems, amongst British-South Asian communities has suggested that some members of the Pakistani community in the Greater Manchester area put greater trust in advice that comes from a General Practitioner (GP), as opposed to other information sources, such as letters or leaflets from government agencies.^13,14^ Therefore, in the breast-screening context, it is important to explore how such GP-patient relationships in the Pakistani community can be utilized to improve access to breast screening.

The literature has noted a need for studies into how different ethnic groups understand cancer and how to improve screening uptake.^12^ Research that has attempted to understand inequalities in the uptake of breast screening mostly does so from a ‘South Asian’ perspective, and the distinct ethnicities of Indian, Pakistani, Bangladeshi, Afghani, Nepalese, Bhutanese, Maldivian and Sri Lankan women tend to be treated as one homogenous group. Similarities exist among people from the South Asian community, but lived experiences of British-Pakistani women – which impact their health-seeking behaviours – need to be considered individually, particularly given their lack of engagement with breast screening.^15^ Pakistani women navigate different barriers owing to their religious beliefs, cultural practices and the kinship networks in Pakistani communities, which impact their decisions.^16^ Moreover, there are significant socioeconomic disparities between South Asians living in the UK, with Pakistanis more likely to live in deprived areas with lower income, less education and increased racial discrimination.^17^

The current study aims to extend the existing literature by exploring in depth the role played by British-Pakistani communities’ knowledge of breast cancer and breast cancer screening, including the role of primary care.

## Methods

### Study design and participants

This study was a secondary analysis of qualitative data originally used in studies published elsewhere.^15,18^ In the primary study, 19 semi-structured interviews with British-Pakistani women residing in East Lancashire, UK, were conducted in 2018–2019. The participants had all moved to the UK from Pakistan. Twelve women were of breast-screening age, five were under 50, and two chose not to report their age. All women of screening age reported to have attended at least one screening appointment.

East Lancashire was selected due to the presence of a dense Pakistani community in the region, and the overall uptake of screening in the entire population of Lancashire (60.6%) falling below the National Health Service target^19^ of 70%. Participants were recruited via a community event organized in East Lancashire, where information and advice about breast cancer were provided. At this event, the aim of the study was also explained, and women were provided with information sheets and contact information of the research team if they were interested in participating. Recruitment was further supported by community contacts who disseminated information about the study in the area and provided the research team’s contact information to women interested in participating.

Of the 19 interviews conducted in the primary study, 18 transcripts were used for this analysis, as the 19^th^ was not audio-recorded or transcribed. The 18 interviewees are identified below as P-1 to P-18.

### Data collection

One-to two-hour semi-structured interviews were conducted in either the participants’ homes or a private space in a community centre. Only participants P-1, P-3, P-4 and P-17 conducted their interviews in English. Of the other 14 interviews, in 13 cases a National Health Service-approved interpreter was appointed, who translated in Urdu and Punjabi, and in the 14^th^ case, P-10, the interview was partly in English and partly interpreted by the participant’s daughter. Before the interviews, the interpreter met with the researcher to run through what was going to be asked and how the interview would run. The interpreters worked within strict guidelines, so during the interviews they only translated, and minimized any side conversations. The interviews followed a topic guide focused on the NHSBSP’s invitation letter, understanding of screening and the screening process, the role of the GP and community and what could be improved.

### Secondary data analysis

This study was a secondary data analysis of a previously collected data set. The analytic techniques applied were similar to the primary analysis. The epistemology of a researcher informs the design of the research methods and the process of theorizing meaning from data.^20^

This reanalysis highlighted aspects of data that were not the focus of the primary analysis, leading to new knowledge production.

Braun and Clarke’s approach to thematic analysis was followed, beginning with reading all 18 transcripts thoroughly, to become familiar with the data.^20^ Thereafter, an iterative process began, involving coding of data on NVivo12. Notes on NVivo12 were taken side-by-side to add reflexivity to the analytic process, and to keep track of coding choices.

Codes were then categorized. Codes with commonalities between them were grouped together to make data sorting easier. These categories were then used to establish preliminary themes, which were then tested for coherence. Their extracts were read again to judge whether there was a pattern forming under that theme, and if the various themes were distinct from each other. This was a cyclical process, involving discussions between HMK and KAR, until the researcher was confident that distinct themes had been succinctly established. Finally, the themes were named to immediately explain what they address, and in relation to words used by participants.

## Results

Data were grouped into three key themes:

1. Women’s knowledge of breasts and breast cancer
2. Role of primary care
3. Community engagement

We discuss the three major themes, and their respective subthemes, below.

### Theme 1: women’s knowledge of breasts and breast cancer

#### Subtheme 1(a): women’s understanding of their own bodies

 Participants were asked why they think women hesitate about attending screening. They replied that women feel uncomfortable both with undressing from the waist up and with talking about the female body.

The participants said Pakistani women were deeply unaware of the mechanisms of their own bodies, which meant making choices for their health difficult. This lack of knowledge extended beyond breast cancer to basic biology, such as periods or pregnancy. One participant shared:

My husband asked me first time, when I’m pregnant, how babies come out. I don’t know. I have never seen. Really, I’m telling the truth, I don’t know which way baby is coming (P-17).

Another participant expressed confusion over whether it was normal to have ‘breasts of different sizes’ (P-1) and whether breast size affects breastfeeding.

Modesty and privacy are valued highly within the Pakistani culture. This wish for privacy extends to discussions around the female body, making it ‘a difficult subject’ (P-14). Due to gaps in understanding, there is a fear about matters of the body. As one participant said:

Because it’s such a private matter and we don’t talk about it openly and we don’t show our private parts to anyone like that … It’s just a fear, maybe because you don’t know your body much (P-1).

#### Subtheme 1(b): women’s perception of breast cancer

 Some participants explained that their cultural context restricts opportunities to learn about preventive lifestyles or to hear about the hopeful side of cancer, for example, that early detection leads to better outcomes. Instead, breast cancer remains a threat in the community that is not spoken of, and that fear stops women from engaging with screening. This lack of engagement also includes any conversation that could have ‘cancer’ and ‘breast’ attached to it. A participant explained:

Breast cancer is a very scary thing … a life-threatening kind of thing. So sometimes people … don’t want to know that information about it, or the worry, or contact, or think ‘I’ve got it’ (P-12).

The disease’s reputation makes receiving an invitation to screening stressful. Upon receiving a letter, some women described not opening it, being scared, or even feeling they could not discuss the invitation in their own homes. As a participant described, via her interpreter:

She’ll put the letter to one side … Even with her husband there are certain things she won’t discuss (P-14).

Participants described breast cancer as ‘the big killer in the community’ (P-2), a ‘silent killer’ (P-5) and something shameful for a woman. This feeling of shame was associated with the fact a diagnosis of breast cancer was perceived as a loss of womanhood*,* particularly by the older generation:

My Mum, back home, she would not say ‘cancer’ … Maybe because the perception is that you lose your hair, you lose your breasts, or your womanhood. It’s not to be talked about (P-1).

Several participants elaborated that shame and embarrassment affects women engaging with screening, since they fear they could have cancer as well, and then face cultural ramifications. One participant explained that for women diagnosed with breast cancer, the norm is silence:

Try to keep a secret in the start. When it’s towards the end, when they know that … this is spread everywhere, then they might open up a little bit, about their disease and what’s happened to them (P-15).

### Theme 2: role of primary care

#### Subtheme 2(a): general practitioner endorsement of breast screening

 Several participants described the sense of comfort and trust they have with their GP. From their perspective, the role of a GP is honourable and important in the community, so the GP’s opinion is trusted and highly valued. As one participant said, ‘[Women] know the doctor [GP], and if the information is given to them by the doctor, that’s fine’ (P-12).

However, when asked about their local GP’s role in engaging with them about breast screening, all but one participant said that there had been no such discussion. The participants felt that the way the breast-screening invitation is currently sent out – that is, a letter from the NHSBSP – feels separate from their usual health care. This made several women unsure of where the invitation had come from and potentially led to the women either ignoring the invitation or regarding it as unimportant. Indeed, one participant explained how mammographers or invitation letters are almost ‘outsiders’ (P-12), while a GP is at the heart of a community and a professional.

According to participants, if their GP endorsed breast screening, this could potentially encourage more women to attend screening:

If they receive a letter just from [the NHSBSP] … maybe they don’t put that much attention and it is just a letter … If you put through the GP, that’s a bit more important (P-16).

#### Subtheme 2(b) primary care as trusted source of information dissemination

 Some participants said they had been offered no breast cancer literature inside their local primary care clinic and had seen no posters in their local primary care centres highlighting breast cancer. Participants believed that more posters in clinics would increase the community’s familiarization with breast cancer and screening.

One participant added that the local primary care centre itself can act as a safe space. If a nurse or a GP is talking to women about breast health, this is a private conversation, making any shame or any stigma attached to the disease irrelevant since the women are within the safe walls of a clinic. It also allows women to ask questions without any embarrassment.

The feeling amongst the participants was that a small discussion with a nurse or a doctor, about why it is a good idea to attend screening, would be extremely beneficial:

If the doctors are playing more of a role in this, and making you aware, then obviously people tend to say, ‘Oh, that is my own doctor.’ … Then there’s more chance of people going [to the screening] ‘cause they think that the doctor is part of [the screening programme]’ (P-12).

### Theme 3: community engagement

A concept reiterated in all interviews was that of the tightknit bond Pakistani communities tend to have. The community prides itself on looking after each other and rallying together, particularly in times of need: ‘Even though we’re not blood-related, we see ourselves as a family’ (P-7).

Participants felt that this community spirit could be harnessed in the fight against breast cancer. One idea was having community open days, with a mix of health professionals and local women who had experienced screening and/or breast cancer community engaging with other women about breast health and screening. One participant explained:

There’s a lot of people who want to help this cause … who want to spread the word, because it’s a very beneficial kind of thing (P-2).

Another said:

If you were to hold such an event at a school twice in the year, that information would disseminate quite quickly … Those mums then would be able to transfer that information to other mums (P-14).

One participant suggested that some women perceive screening as running counter to their religion. They believe their faith in God to heal them supersedes all, including medical intervention, and they prefer to engage with faith-based healers. This participant went on to say if she had the chance to talk to such women, she would give them a different perspective:

If you have a cut and it’s bleeding, you need to do something to stop the bleeding. God will give you the power to heal, but you need to get stitches to stop the blood (P-16).

The participant said she would be happy to tell other women that looking after oneself through preventive measures like breast screening also aligns with their faith, as leading a healthy lifestyle is encouraged in Islam. That is because this would stop them from suffering later.

Participants stressed the importance of involving all the family in community outreach. Participants noted that while breast cancer may affect women more than it does men, many men in the community are translators, caretakers or supporters, and therefore have a key role in promoting breast screening. After all, when a woman develops breast cancer, it affects the entire family. As one participant observed: ‘It’s kind of affecting everybody in a lot of ways. You’re saving your kids, your husband, your mum, your sisters’ (P-8).

Some participants suggested community leaders distribute leaflets and information cards in nurseries, clinics, and other locations that women frequent. Participants added that tailored outreach involving mosques and men would also prove beneficial:

Get the mosques to encourage the husbands to make sure they send the women to these different venues you might be running the information at (P-14).

Many participants were determined that the future be different for their daughters. To achieve that, the community must work together: ‘We have to prepare for our next generation’ (P-16).

## Discussion

This study offers important insights surrounding breast cancer and screening in the British-Pakistani community. This leads us to suggest three main targets for future interventions to improve access to breast screening for British-Pakistani women.

First, there should be co-produced strategies to increase knowledge of breasts and breast screening. There is a lack of knowledge among Pakistani women (particularly older women) of the female body. Previous studies have reported that within Pakistani communities there is a hesitancy to even say the word ‘breast’ and it is at times replaced with ‘chest’, which causes misconceptions of the aetiology of the disease.^9^ Our analysis echoes those findings, with some women feeling uncomfortable with the word ‘breast’.

To improve uptake of breast screening, previous studies have noted the importance of increasing knowledge of screening itself.^15,21^ Although this is no doubt true, improving Pakistani women’s awareness and understanding of breast screening may also need to incorporate an initial introduction to the anatomy and physiology of breasts. This could include an education campaign explaining the functions of the breasts, that different breast shapes and sizes are normal, and the need for breast screening (similar to the Scottish breast-awareness campaign led by actress Elaine C. Smith).^22^ These knowledge-building efforts could include leaflets, co-produced with British-Pakistanis, translated into multiple community languages and handed out at community events.

Second, there should be greater collaboration with local GPs to support women to make informed choices about screening. GPs’ role in preventive care could be expanded, with the appropriate resources provided. This could include having informational leaflets in local health centres. Also, primary care facilities could be advised when NHSBSP invitations are sent out, so that GPs can encourage women, when they come in for regular appointments, to consider screening.

Previous studies have discussed how some Pakistani women delay seeking help and only do so when the pain of breast cancer becomes unbearable.^10^ The cultural taboo surrounding the illness leads women to think of a potential diagnosis as bad luck and that breast cancer is contagious.^11^ Some women reportedly do not tell their family members or close friends about the illness, preferring to carry the burden alone and using *chaddars* (long drapes) to cover any changes in appearance. This is all due to the fear of local gossip and possible social isolation.^9,11^ Primary care could alleviate this fear, with GPs explaining screening, the procedure of screening and its benefits, including early detection. Involving primary care to increase uptake of screening has been recommended previously.^23^ Additionally, a study based in New Zealand found that medical professionals talking Pakistani women through educational material has been effective in improving uptake of breast and cervical screening.^21^

Third, there should be community engagement involving GPs and community leaders, to inform everyone – not just screening-age women – about breast cancer and screening. Health-related interventions often fail because policy reform mostly focuses on individual behavioural choices rather than accounting for the social, economic and political factors that play into decisions individuals make.^24^ The participants in this study felt that the community offered an opportunity to improve understanding and access to breast screening. Community groups have been successfully used in interventions, to encourage camaraderie and provide extra support.^23,25,26^ Community events co-produced by health professionals and community groups can be helpful. At the events, health professionals could inform women of screening and explain how a breast cancer diagnosis is not necessarily a death sentence.

Women who have attended screening could provide an important perspective in co-designing and taking part in awareness-related interventions. Such women could talk to those reluctant to attend a screening – taking into account their faith and culture – and discuss any concerns. Moreover, some participants insisted that outreach cannot be solely designed for women.^21^ Knowledge in the community must be improved as a whole, which includes better-informed men and families. Traditionally, men in Pakistani culture have key roles – for some women, their husbands or fathers could even be making medical decisions on their behalf.

## Limitations

This study has three main limitations. First, it did not include views of women who choose not to attend screening. Their insight into those aspects of NHSBSP that prevent them from attending screening is integral to inform long-lasting improvements.

Second, most interviews involved translation. This meant the participants’ ability to express themselves freely was reduced through someone translating their words and possibly missing information.

Third, this was a secondary analysis using existing data. This meant we were unable to probe the participants for further details on individual points.

## Conclusions

The British-Pakistani community has specific patterns and interactions, which cannot be homogenized under studies simply involving ‘South Asians’. A proportionate universalism approach is essential if we are to address inequalities in screening participation.^27^

Future research could focus on studying young British-Pakistani women’s perspectives on understanding their bodies and combatting breast cancer. Research could draw comparisons across generations, to gauge what is most likely to lead to potential improvements.

The strategies outlined in this study could also be extrapolated to other minority communities within the UK, encouraging learning across different cultural groups and amplifying efforts to increase breast-screening uptake. While some barriers to breast screening are different across cultural groups, many are shared, and inclusive interventions to encourage screenings may be beneficial.^28^
